# Complete mitochondrial genome of the three Colombian Caribbean loggerhead sea turtles, using next-generation sequencing

**DOI:** 10.1080/23802359.2018.1450664

**Published:** 2018-05-21

**Authors:** Javier Hernández-Fernández, David Arley Delgado Cano

**Affiliations:** Universidad de Bogota Jorge Tadeo Lozano, Ciencias Biológicas y Ambientales, Bogota, Cundinamarca, Colombia

**Keywords:** *Caretta caretta*, mitogenome, Illumina next-generation sequencing

## Abstract

The loggerhead turtle, *Caretta caretta* (Linnaeus, 1758), is an endangered sea turtle in Colombian Caribbean beach. In the present study was sequenced the complete mitochondrial DNA of three loggerhead turtles using Illumina next-generation sequencing (NGS). The average nucleotide frequency was A: 35% T: 26%, C: 27% and G: 12%. This genome provides knowledge to the study of genetic variations and evolution of mitochondrial genomes of *C. caretta*. The sequences were deposited at the GenBank database under the accession number MF554690.1, MF579504.1 and MF579505.1.

The loggerhead turtle is a marine turtle belonging to the Cheloniidae family, order Testudines. Loggerhead turtle is a species widely distributed in the tropical and Central Atlantic and Indo-Pacific region (Lancheros-Piliego and Fernández 2013). Due to the population decrease of this species in the oceans around the world, generated by anthropic actions such as egg consumption, use of shells, commercial fishing and destruction of natural habitats, among others (Lutcavage et al. 1997), survival of loggerhead turtles is considered Vulnerable A2b globally (IUCN 2017) and Critically Endangered A2cd; D in Colombia (Páez et al. 2015). To generate conscience and contribute to its conservation, phylogenetics, genetics of populations and evolution analyses have been carried out using mitochondrial DNA. Blood samples from three individuals of loggerhead turtle were obtained from the CEINER Oceanarium in San Martin de Pajares Island, Cartagena (10°11′N, 75°47′W) following the Dutton methodology (Dutton 1996). The blood samples were used for total RNA extraction using RNeasy Mini Kit (Quiagen, Hilden, Germany). For mRNA library preparation, we use a TruSeq RNA Library Prep Kit v2 according to manufacturer’s instructions (Illumina, San Diego, CA, USA). The quality control of generated libraries was done using the 2100 bioanalyzer (Agilent, Santa Clara, CA, USA). RIN values (RNA integrity number) of 7.5 were obtained. The library was paired-end sequenced using Hiseq 2000 Platform (Macrogen, Inc, Korea) (Hernández-Fernández et al. 2017). The quality of cleaned raw reads was verified with the fastQC program (http://www.bioinformatics.bbrc.ac.uk/projects/fastqc/). Using the Local Basic Alignment Search Tool (BLASTn) the contigs of each transcriptome were filtered to establish the complete mitogenome of each individual by aligning the local data obtained from the sequencing against a mitochondrial genome of *C. caretta* previously reported in GenBank (NCBI) (access number: NC_016923. 1). For each consensus sequence, paired alignments were made with each of the 37 genes encoded by the mtDNA (reported in GenBank) using the BLAST refseq_genomic tool (NCBI). The phylogenetic tree was done using software MEGA 6 (Tamura et al. 2013) using the maximum likelihood algorithm (Guindon and Gascuel, 2003) (Figure 1). The three complete mitogenomes of loggerhead turtles were annotated, they comprised 16,633, 16,461 and 16,446 bp, composed for 13 protein-coding genes, 22 tRNA, 2 rRNA and a non-coding control region. These mitogenomes have a typical order of terrestrial, freshwater, and marine turtles (Drosopoulou et al. 2012; Duchene et al. 2012) and vertebrates in general (Boore, 1999). Our analysis of the loggerhead mitogenomes is the first report in the Colombian Caribbean, generating new basic information for studies at a genetic level and contributing to management and conservation suitable plans for this species.

**Figure 1.**
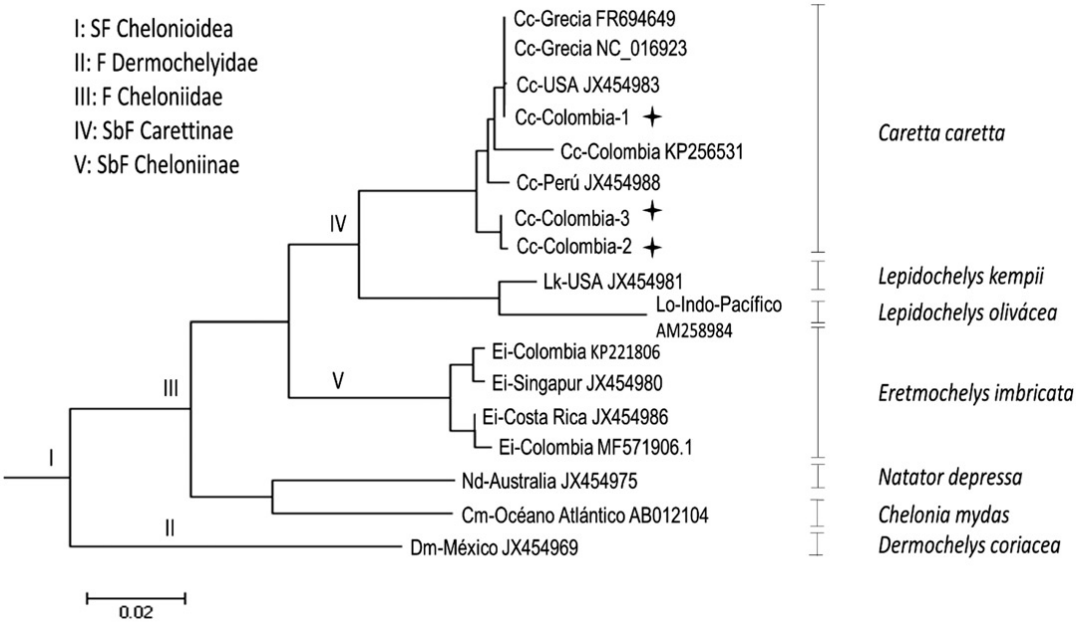
Philogenetics tree obtained using the algorithms máxima verosimilitud (MV), used complete mitogenomes. The topology of the tree shows correct associations between turtles forming corresponding relationships between subfamilies (SbF), families (F), and superfamilies (SF).
